# Association between Emotional Eating and Frequency of Unhealthy Food Consumption among Taiwanese Adolescents

**DOI:** 10.3390/nu13082739

**Published:** 2021-08-10

**Authors:** Chung Bui, Li-Yin Lin, Chih-Yi Wu, Ya-Wen Chiu, Hung-Yi Chiou

**Affiliations:** 1 Ph.D. Program in Global Health and Health Security, College of Public Health, Taipei Medical University, 250 Wuxing St., Taipei City 110, Taiwan; d537108005@tmu.edu.tw; 2 Department of Health Communication and Education, Quang Ninh Provincial Center for Disease Control (CDC) in Vietnam, 651 Le Thanh Tong St., Bach Dang Ward, Ha Long 01108, Vietnam; 3 Master Program in Applied Epidemiology, College of Public Health, Taipei Medical University, 250 Wuxing St., Taipei City 110, Taiwan; jlin11025@gmail.com; 4 School of Public Health, College of Public Health, Taipei Medical University, 250 Wuxing St., Taipei City 110, Taiwan; 5 Department of Leisure Industry and Health Promotion, National Taipei University of Nursing and Health Sciences, 365 Mingde Road, Beitou District, Taipei 11219, Taiwan; 6 Institute of Population Health Sciences, National Health Research Institutes, Zhunan Town, Miaoli County 35053, Taiwan; jmps7129@gmail.com; 7 Master Program in Global Health and Development, College of Public Health, Taipei Medical University, 250 Wuxing St., Taipei City 110, Taiwan

**Keywords:** emotional eating, unhealthy food consumption, risk factors, adolescents, public health

## Abstract

Emotional eating is one factor that increases the consumption of unhealthy food. This study aimed to investigate the association between emotional eating and frequencies of consuming fast food, high-fat snacks, processed meat products, dessert foods, and sugar-sweetened beverages (SSBs) in adolescents. The baseline survey data (2015) from the Taiwan Adolescent to Adult Longitudinal Study (TAALS) were fitted into multivariate logistic regression models adjusted for sex, school type, Body Mass Index (BMI), eating while doing something, nutrition label reading, skipping breakfast, smoking, binge drinking, sedentary lifestyle, physical activity, peer and school support, and parental education level. Among the 18,461 participants (48.5% male and 51.5% female), those exhibiting emotional eating were more likely to consume fast food (Odds ratio (OR) = 2.40, 95% Confidence interval (CI): 2.18–2.64), high-fat snacks (OR = 2.30, 95% CI: 2.12–2.49), processed meat products (OR = 1.92, 95% CI: 1.78–2.08), dessert foods (OR = 2.49, 95% CI: 2.31–2.69), and sugar-sweetened beverages (OR = 1.83, 95% CI: 1.70–1.98). Factors that were positively associated with unhealthy food consumption included eating while doing other activities, binge drinking, smoking, and sedentary lifestyle. Among all the covariates, nutrition label reading was the only factor that was inversely associated with frequent unhealthy food consumption. Sex and school type may moderate the effect of emotional eating on the frequent consumption of specific unhealthy food groups. In conclusion, adolescents with high emotional eating were more likely to report frequent consumption of unhealthy foods in Taiwan. Our findings showed that male participants appeared to consume fast foods, high-fat snacks, processed meat, and SSBs more often and dessert foods less often than females. Future longitudinal studies are recommended for understanding the causal relationship between emotional eating and unhealthy food consumption.

## 1. Introduction

According to the World Health Organization (WHO) Nutrient Profile for South-East Asia Regions, unhealthy foods are foods high in energy, sodium, and sugar content and low in nutrients such as protein, essential fatty acids, vitamins, minerals, and fiber [1]. Ultraprocessed foods (UPFs) are a subset of unhealthy foods and are defined as industrially formulated foods with the same nutritional characteristics as those listed above for unhealthy foods [2]. UPFs often contain either food substances that rarely appear in the home kitchen (such as high-fructose corn syrup, hydrogenated or inter-esterified oils, and hydrolyzed proteins) or food additives with the purpose of imitating the natural flavors or increasing palatability (such as flavors; colorants; emulsifiers; emulsifying salts; artificial sweeteners; thickeners; and antifoaming, bulking, carbonating, foaming, gelling, and glazing agents) [2].

A variety of studies have shown strong correlations between unhealthy food consumption and noncommunicable diseases (NCDs), such as obesity, breast cancer, rectal colon cancer, ischemic heart diseases, diabetes, stomach cancer, and hypertension [2,3,4,5,6,7,8,9]. In particular, being overweight or obese is strongly associated with overconsumption of energy-dense food and physical inactivity. Globally, 39% of adults were overweight and 13% were obese in 2016. Being overweight or obese are major risk factors for NCDs, such as cardiovascular diseases, diabetes, musculoskeletal disorders, and some cancers [10]. In addition, excessive consumption of salt is positively associated with cardiovascular diseases, the leading cause of death [11]. High sodium intake was also attributed to 4.1 million annual deaths globally according to the WHO [12]. In addition to the diseases listed above, recent population-based, cross-sectional, and cohort studies also found that higher intake of UPFs may lead to a higher risk of gastrointestinal disorders, asthma, frailty, and depression [13,14,15,16].

Unhealthy food intake has become a serious global health concern. UPFs have become a major part of diets in almost every corner of the world because they are widely available, cheap, and aggressively marketed [17,18,19]. At the individual level, consumption behaviors are also influenced by several factors, including socioeconomic determinants (such as sex, age, education level, income) [20], nutritional knowledge, and individual health status (such as physical and psychological well-being) [21].

Other research has shown that adolescents consume unhealthy food more than other age groups [20,22,23,24,25]. Emotional eating, defined as increased eating in response to negative or positive emotions, is a factor associated with the increased consumption of unhealthy foods [26,27,28,29,30,31]. Emotional eating was initially associated with depression and negative emotions, and several questionnaires were developed and validated to measure emotional eating [32,33,34,35,36]. Experimental studies have explained the link between negative emotions and increased food consumption [31], but recent studies have shown that positive emotions have more influence over food consumption than negative emotions [37].

In Taiwan, according to analysis from the Nutrition and Health Surveys in Taiwan, UPF consumption among Taiwanese adolescents increased between 1993 and 2011 [38]. Additionally, other studies in Taiwan have shown that the consumption of sweetened beverages gradually increased and became more common among adolescents [39,40]. One serious health-related concern about unhealthy eating behaviors is their association with depression [41]. Based on existing evidence, we proposed that emotional eating may relate to a higher risk of frequent unhealthy food consumption. In this population-based, cross-sectional study, we aimed to assess the association between emotional eating and frequency of unhealthy food consumption among Taiwanese adolescents and the potential influencing factors associated with emotional eating.

## 2. Materials and Methods

### 2.1. Study Population

The present study used data from the Taiwan Adolescent to Adult Longitudinal Study (TAALS), which was a school-based, nationally representative, longitudinal survey conducted between 2015 and 2019 [42]. The baseline survey for the TAALS was conducted in 2015. Between 2015 and 2019, the survey was repeated three times to observe changes in health behaviors among participants over time. A multistage stratified sampling approach with probability proportional to size sampling was applied to obtain a nationally representative sample of adolescents. Participants were first-year students in junior high school, senior high school, and vocational high school in 173 nationally representative sample schools. We excluded students who did not have a signed parental consent form and those who did not thoroughly answer questions regarding eating behavior. Among the selected 18,461 records, 6799 (36.8%) were junior high school students (Grade 7, mean age = 13), 4780 (25.9%) were senior high school students (Grade 10, mean age = 16), and 6882 (37.3%) were vocational high school students (Grade 10, mean age = 16). The questionnaire used for the TAALS was developed through a systematic review of large-scale international youth studies. Questions from the questionnaire were adapted from existing questionnaires used in other nationally representative health surveys, including Add Health (The National Longitudinal Study of Adolescent to Adult Health); The Global Youth Tobacco Survey (GYTS) by the WHO; the Measuring Bullying Victimization, Perpetration, and Bystander Experiences Assessment Tools compiled by the U.S. Centers for Disease Control and Prevention; and The Youth Risk Behavior Surveillance System (YRBSS) survey [43,44,45,46]. Our questionnaire was designed to specifically target young adolescents between the ages of 11 to 18 years old and included questions about demographic information, lifestyle and physical activity, substance use, dietary behaviors, mental health, violence-related behaviors and experiences, sexual behaviors and attitudes, and social support. An expert validity test was also conducted to evaluate the reliability and validity of our questionnaire, and each question from the questionnaire achieved a content validity index (CVI) score between 0.9 and 1.0. The TAALS was a cohort study funded by Taiwan’s Health Promotion Administration (HPA). Our access to the TAALS dataset was granted by the HPA for data analysis and publication purposes. The baseline survey for the TAALS was conducted in 2015, and the TAALS cohort was followed until 2019 with three follow-up surveys. The TAALS study was approved by the Joint Institutional Review Board of Taipei Medical University, Taiwan (TMU-JIRB-201410043). The original data collection for the TAALS study and our subsequent analysis of the survey results were both approved by the Joint Institutional Review Board of Taipei Medical University, Taiwan (TMU-JIRB-201410043).

### 2.2. Frequent Unhealthy Food Consumption

We selected the unhealthy food groups that are often associated with health problems or health risk factors, including fast food, high-fat snacks, processed meat products, dessert foods, and sugar-sweetened beverages. The TAALS questionnaire included five items about unhealthy foods and their consumption frequency: In the past week, (1) how many times did you eat fast foods (i.e., French fries, fried chicken, fried chicken, pizza, burger, instant noodles)? (2) How many times did you eat high-fat snacks (i.e., potato chips, croissants, fried egg wrap, scallion pancakes)? (3) How many times did you eat processed meat products (i.e., sausage, hot dog, ham, bacon)? (4) How many times did you eat dessert foods (i.e., candy, chocolate, cream, pudding, cookies)? (5) How many times did you drink sugar-sweetened beverages (i.e., bubble milk tea, fruit juice drinks, soft drinks, sports drinks, sugary tea drinks, Yakult)? Previous cohort studies defined high/frequent consumption of a certain food as greater than 2 times per week [47,48,49]. Therefore, we adopted similar cutoff values and classified the consumption of a certain food group as “low” (<3 times per week) and “high” (≥3 times per week). For each unhealthy food group, all questions reached a good internal consistency (Cronbach’s α = 0.73).

### 2.3. Emotional Eating

The questionnaire used in this study was a common measurement tool for emotional eating [33,34,35,36,37]. Frequencies of eating in response to positive and negative emotions were assessed by two questions: In the past week, (1) how many times did you eat to reward yourself or celebrate? (2) How many times did you console yourself when feeling sad or stressed by eating? These questions were scored as follows: 1 = never, 2 = 1–2 times, and 3 = three times or higher. The two scores were summed to calculate the participant’s emotional eating score. Based on the participant’s emotional eating score, ranging from 1 to 6, we categorized participants with a score of 4 or above as “highly sensitive to emotional eating” (high EmE) and participants with a score under 4 as “less sensitive to emotional eating” (low EmE). The two questions addressing emotional eating frequency showed good internal consistency (Cronbach’s α = 0.72).

### 2.4. Individual Factors, Other Eating Behaviors, Lifestyle, and Social Determinants

Certain factors, based on a literature review [28], were used to determine the potential covariates and moderators for emotional eating. Individual factors include sex, Body Mass Index (BMI), and type of school (junior high school, senior high school, or vocational high school). According to the WHO Expert Consultation for Appropriate Body Mass Index for Asian Populations [50], the Taiwan Ministry of Health and Welfare defines BMI status as follows: “underweight”, BMI < 18.5; “normal”, 18.5 ≥ BMI < 24; and “overweight”, BMI ≥ 24 [51]. We categorized the frequency of skipping breakfast, eating while doing other activities, and reading nutrition labels based on how many times the participants engaged in the activity in the past week: “low” (0–2 times/week) and “high” (≥3 times/week). Cigarette smoking was dichotomized (yes/no) from the self-reported frequency during the past month. For the definition of binge drinking behavior in adolescents, we used the criterion of ≥5 drinks on one occasion for at least one day during the past month [52,53,54]. As for sedentary behavior, similar to a recent study [55], we classified screen-time-based sedentary behavior as spending ≥2 h/day watching TV, playing video games, or using a computer/mobile phone. According to the WHO Global Recommendations for Physical Activity for Health [56], we defined physical activity as physical activity for ≥60 mins/day occurring ≥3 days/week in the past week. Peer support was measured based on agreement with four statements such as “my classmates/friends care about what happens to me” (range 4–16, cutoff value of ≥8, Cronbach’s α = 0.90). School support was based on response to six statements such as “my school is a good place to be” (range 6–24, cutoff value of ≥12, Cronbach’s α = 0. 88). Parental education level was defined using the highest education level achieved and categorized as follows: (1) junior high school graduate or below, (2) senior high school graduate, (3) university graduate.

### 2.5. Statistical Analysis

To compare students’ characteristics and frequent unhealthy food consumption, we performed a chi-square test for categorical variables. A logistic regression model was used to assess the association between emotional eating and frequent unhealthy food consumption. The model was adjusted for sex, school type, BMI, eating while doing something, nutrition label reading, skipping breakfast, smoking, binge drinking, sedentary lifestyle, physical activity, peer and school support, and parental education level. All analyses were performed using SPSS version 16.0, and *p* < 0.05 was considered significant.

## 3. Results

### 3.1. Characteristics of Participants and Unhealthy Food Consumption

Table 1 shows the characteristics of the participants and the frequencies of unhealthy food consumption. Among the 18,461 participants, 8953 (48.5%) were male and 9508 (51.5%) were female. There were 6882 (37.3%) junior high school students, 4780 (25.9%) senior high school students, and 6799 (36.8%) vocational high school students. There were few missing values due to incomplete or unusable responses to survey questions. Half of the participants had normal BMI (53.0%), and there were more underweight than overweight participants (29.7% vs. 17.3%). Almost one-third of participants responded with high EmE (31.8%). Frequencies of unhealthy food consumption, in order from the most to the least consumed, were 60.1% for sugar-sweetened beverages (SSBs), 46.7% for dessert foods, 29.8% for processed meat products, 26.5% for high-fat snacks, and 16.0% for fast foods. Only 46.9% of the participants reported reading nutrition labels often, and more than half of the participants reported eating while doing something (52.4%). Only 11.3% of the adolescents reported frequently skipping breakfast. As for lifestyle factors, more than half of participants (54.3%) engaged in sedentary activities (i.e., watching TV, playing video games, or using a mobile phone for more than 2 h). Only 46.6% of the participants reported frequently doing physical activities. There were few participants who were smokers and binge drinkers in our study (5.6% and 6.5%, respectively). Most participants reported receiving good peer support (86.0%) and good school support (93.2%). As for parental education, about one-third of the participants reported that their mother (34.1%) and father (35.7%) graduated from university. Compared to males, females were more likely to exhibit high EmE (33.6% vs. 29.8%, *p* < 0.001). Males were more likely than females to report frequent consumption of fast food (19.2% vs. 12.9%, *p* < 0.001), high-fat snacks (28.8% vs. 24.3%, *p* < 0.001), processed meat products (35.5% vs. 24.5%, *p* < 0.001), and SSBs (64.7% vs. 55.8%, *p* < 0.001). However, females were more likely than males to report frequent consumption of dessert foods (49.6% vs. 43.4%, *p* < 0.001).

### 3.2. Associations between Emotional Eating, Personal, Behavioral, and Socioeconomic Factors and Frequency of Unhealthy Food Consumption

#### 3.2.1. Frequency of Fast Food Consumption

In the adjusted model (Table 2), participants with high emotional eating were more likely to report frequent fast food consumption compared to those with low emotional eating (OR = 2.40, 95% CI: 2.18–2.64). Males were more likely to report frequent fast food consumption compared to females (OR = 1.63, 95% CI: 1.48–1.80). Participants who indicated eating while doing something were more likely to report frequent fast food consumption compared to participants who did not eat while doing other things (OR = 2.05, 95% CI: 1.85–2.28). Participants with binge drinking behavior were more likely to report frequent fast food consumption compared to participants who did not binge drink (OR = 1.29, 95% CI: 1.09–1.53). Participants who smoked were more likely to report frequent fast food consumption compared to their nonsmoking counterparts. Participants with sedentary behavior were more likely to report frequent fast food consumption compared to non-sedentary participants (OR = 1.59, 95% CI: 1.44–1.76). Finally, participants who read nutrition labels were less likely to report frequent fast food consumption compared to participants who did not read nutrition labels (OR = 0.91, 95% CI: 0.83–0.99).

#### 3.2.2. Frequency of High-Fat Snack Consumption

In the adjusted multivariate regression model (Table 2), participants with high emotional eating were more likely to report frequent consumption of high-fat snacks compared to their low emotional eating counterparts (OR = 2.30, 95% CI: 2.12–2.49). Participants exhibiting eating while doing something behavior were more likely to report frequent consumption of high-fat snacks compared to participants who did not eat while doing other things (OR = 2.28, 95% CI: 2.09–2.47). Participants with sedentary behaviors were more likely to report frequent high-fat snack consumption compared to those without sedentary behaviors (OR = 1.41, 95% CI: 1.30–1.53). Lastly, participants who read nutrition labels were less likely to report frequent consumption of high-fat snacks compared to participants who did not read nutrition labels (OR = 0.87, 95% CI: 0.80–0.94). However, binge drinking and smoking were not significantly associated with frequent high-fat snack consumption.

#### 3.2.3. Frequency of Processed Meat Product Consumption

In the adjusted model (Table 2), participants with high emotional eating were more likely to report frequent processed meat product consumption compared to those with low emotional eating (OR = 1.92, 95% CI: 1.78–2.08). Male participants were more likely to report frequent processed meat product consumption compared to female participants (OR = 1.71, 95% CI: 1.59–1.85). Participants exhibiting eating while doing something behavior were more likely to report frequent consumption of processed meat products compared to participants who did not eat while doing other things (OR = 1.72, 95% CI: 1.59–1.86). Participants exhibiting sedentary behavior were more likely to report frequent consumption of processed meat products compared to participants without sedentary behaviors (OR = 1.33, 95% CI: 1.23–1.44). Participants with binge drinking behavior were more likely to report frequent consumption of processed meat products compared to participants who did not binge drink (OR = 1.21, 95% CI: 1.04–1.40). However, nutritional label reading was not significantly associated with frequent consumption of processed meat products.

#### 3.2.4. Frequency of Dessert Food Consumption

In the adjusted model (Table 2), participants with high emotional eating were more likely to report frequent dessert food consumption compared to those with low emotional eating (OR = 2.49, 95% CI 2.31–2.69). Participants exhibiting eating while doing something behavior were more likely to report frequent consumption of dessert foods compared to participants who did not eat while doing other things (OR = 2.08, 95% CI 1.94–2.24). Participants exhibiting sedentary behaviors were more likely to report frequent consumption of dessert foods compared to participants without sedentary behaviors (OR = 1.19, 95% CI 1.11–1.28). Participants with binge drinking behavior were more likely to report frequent consumption of desert foods compared to participants who did not binge drink (OR = 1.24, 95% CI: 1.07–1.44). Lastly, male participants were less likely to report frequent dessert food consumption compared to female participants (OR = 0.78, 95% CI: 0.73–0.84). However, smoking and nutrition label reading were not significantly associated with dessert food consumption (OR = 1.19, 95% CI 1.11–1.28).

#### 3.2.5. Frequency of Sugar-Sweetened Beverage (SSB) Consumption

The full regression model showed that participants with high emotional eating were more likely to report frequent SSB consumption compared to those with low emotional eating (OR = 1.83, 95% CI: 1.69–1.98). Male participants were more likely to report frequent SSB consumption compared to female participants (OR = 1.43, 95% CI: 1.33–1.54). Participants exhibiting eating while doing something behavior were more likely to report frequent consumption of dessert foods compared to participants who did not eat while doing other things (OR = 2.17, 95% CI: 2.02–2.33). Participants exhibiting sedentary behaviors were more likely to report frequent consumption of SSBs compared to participants without sedentary behaviors (OR = 2.17, 95% CI: 2.02–2.33). Lastly, participants who read nutrition labels were less likely to report frequent SSB consumption compared to participants who did not read nutrition labels (OR = 0.82, 95% CI: 0.76–0.88). However, binge drinking was not significantly associated with SSB consumption (OR = 0.98, 95% CI 0.84–1.15).

### 3.3. Stratification Analyses

Table 3 shows the moderating effects of sex and school type (i.e., junior high school, high school, vocational high school) on the association between emotional eating and frequency of unhealthy food consumption. Male students with high EmE from the junior high school group were more likely to consume processed meat products (OR = 1.24, 95% CI: 1.00–1.54) and less likely to consume high-fat snacks (OR = 0.77, 95% CI: 0.62–0.95) than those from the vocational high school group. In addition, male students with low EmE from the senior high school group were more likely to consume fast foods (OR = 1.36, 95% CI: 1.00–1.70), processed meat products (OR = 1.36, 95% CI: 1.16–1.60), dessert foods (OR = 1.18, 95% CI: 1.01–1.38), and SSBs (OR = 1.47, 95% CI: 1.26–1.71) than those from the vocational high school group, male students with low EmE from the junior high school group were more likely to consume fast foods (OR = 1.26, 95% CI: 1.03–1.55) and SSBs (OR = 1.16, 95% CI: 1.00–1.33) and were less likely to consume high-fat snacks (OR = 0.69, 95% CI: 0.58–0.81) and dessert foods (OR = 0.78, 95% CI: 0.67–0.90) than those from the vocational high school group. Lastly, female students with low EmE from the junior high school group were less likely to consume high-fat snacks (OR = 0.76, 95% CI: 0.63–0.91) and dessert foods (OR = 0.63, 95% CI: 0.55–0.73) than those from the vocational school group.

## 4. Discussion

In this study, we assessed the prevalence of emotional eating among Taiwanese adolescents in 2015 and its association with certain types of food groups. Our findings provide a representative model for the frequent consumption of fast foods, high-fat snacks, processed meat products, dessert foods, and SSBs among young adolescents and the influence of complex factors including health behaviors, individual factors, and social determinants. Our work expanded current knowledge regarding emotional eating by revealing several significant risk factors associated with frequent consumption of unhealthy foods. Furthermore, we stratified the participants by sex and school type to examine how these two factors moderate the association between emotional eating and frequent consumption of unhealthy foods.

We found that high emotional eating was associated with frequent consumption of fast foods, high-fat snacks, processed meat products, dessert foods, and SSBs among adolescents. Similarly, other researchers also documented a positive association between emotional eating and consumption of sugary foods, fast foods, high-fat snacks, sweets, and soft drinks [57,58,59]. Adolescence has been characterized by a variety of studies as an emotional period with strong and unpredictable emotional shifts, including not only depression and anxiety, but also exuberance and elation [57]. Difficulties in emotional regulation may drive adolescents to be emotional eaters [60] who tend to consume sweets, salty foods, and other energy-dense foods [61]. Previous studies have investigated how emotions regulate the consumption of unhealthy foods by investigating the association between a high-sugar or a high-fat diet and the release of neurotransmitters such as dopamine [62,63]. In another study, adolescents’ food choices appear to have been influenced by social media, the internet, branding, advertisements, and other factors. The researchers reported that, partially because of these information sources, adolescents’ nutrition knowledge appeared to be limited, and their food environment was frequently dominated by UPFs [64].

Our findings indicated that eating while doing other activities should be considered as a critical risk factor for frequent unhealthy food group consumption. This finding supports the observation that eating while doing other things may be associated with weight gain because unhealthy food intake has been associated with both eating while doing other activities and weight gain [65,66]. Our results also suggested that a sedentary lifestyle is significantly associated with unhealthy food consumption. According to the WHO, a sedentary lifestyle may increase the risk of increased all-cause mortality, cardiovascular diseases, diabetes, obesity, colon cancer, high blood pressure, osteoporosis, lipid disorders, depression, and anxiety [67,68]. Other studies have also demonstrated that a sedentary lifestyle is strongly correlated to obesity and a variety of NCDs such as diabetes, cardiovascular diseases, hypertension, and cancer [53,54].

A processed food is any food that has been altered in some way during preparation. For instance, some common examples of food processing are freezing, drying, canning, or baking. Not all processed foods are unhealthy, but if the processed foods contain high levels of salt, sugar, or fat, then they are considered unhealthy [69]. From a nutritional standpoint, food processing does not refer to transforming an unprocessed food into an unhealthy food or reducing its nutrient content. Indeed, consumption of enriched or fortified foods (i.e., milk fortified with vitamin D and yogurt fortified with probiotics) could be considered beneficial for those who have inadequate intakes of micronutrients such as vitamin A, vitamin C, vitamin D, vitamin E, thiamin, folate, calcium, magnesium, and iron. However, we also need to be careful about processed foods because it is easy to increase salt, sugar, and saturated fat content during food processing [69]. A food should be labeled as an unhealthy food based on its nutrient composition rather than the fact it has undergone processing. In addition, encouraging consumers to read the nutrition labels could help to promote healthy food choices. In our study, nutrition label reading appeared to be a protective factor for the high consumption of fast food, high-fat snacks, and sugar-sweetened beverages. A previous study showed that nutrition label reading may be an important precursor to positive dietary change [70]. Data from the Mexican National Health and Nutrition Survey in 2016 demonstrated a negative association between nutrition label reading and obesity, diabetes, and other chronic conditions [71].

Our findings showed that males appeared to consume fast foods, high-fat snacks, processed meat, and SSBs more often and dessert foods less often than females. These sex differences in frequency of unhealthy food consumption may be due to divergent factors influencing consumption patterns. We observed considerable differences between males and females in terms of emotional eating, lifestyle, nutrition label reading, habit of skipping breakfast, smoking, and binge drinking. In particular, we found that the proportion of males who were overweight (BMI ≥ 24) was significantly higher than that of females. Previous studies proposed that weight status and negative body image may influence eating behaviors [72]. Moreover, comfort food preferences have been previously explored, and the preferences were found to be different among males and females. Males were found to prefer high-fat or high-salt comfort foods (such as steak), while females preferred sweetened snacks (such as chocolate and ice cream) [73]. Lastly, a web-based prospective study called NutriNet-Santé Study showed a higher association between emotional eating and consumption of energy-dense foods in women compared to men. However, significant associations between emotional eating and high-salt fast food and sweetened cream desserts were found only in men [29]. Another study of Belgian children did not show any sex differences in dietary patterns and emotional eating [74]. Overall, males and females appear to be different in terms of the frequency, intensity, and stability of their emotional shifts during the pubertal period [75]. A longitudinal study, with a 15-month follow-up, of adolescents aged between 13 and 17 showed that the levels of instability in negative emotions appeared to remain consistent over time in males, whereas they may become lower (i.e., less unstable) over time in females [76]. Adolescent maturation and individual internal (biological or psychological) and external (socioeconomic and cultural) factors [77] have been considered in a variety of studies as the basis of the sex differences in eating behaviors, food choices, and nutrition strategies. Females appeared to be more health-conscious than males and prefer to control their body weight on a regular basis. On the other hand, previous studies on food choices reported that men seem to prefer fatty meals with a strong taste and enjoy the pleasure associated with eating [78].

The findings reported here expand on previous research showing that young (mean age = 13) and older adolescents (mean age = 16) may be different in how frequently they consume unhealthy food. Among students with low emotional eating, we observed that junior high school students were less likely than older students to consume high-fat snacks and dessert food. Additionally, the positive association between increasing SSB and fast food consumption and being a junior high school student was observed only among males. Previous studies of SSBs [39] and UPFs consumption [38] among Taiwanese adolescents did not find significant differences across age. The observed variance in emotional eating patterns may be mediated by the mood variability of adolescents. A Dutch five-year longitudinal study showed that happiness, anger, and sadness variability continuously decline across adolescence, while anxiety variability increased initially, then decreased, and then increased again towards late adolescence [79]. On the other hand, older adolescents were more likely to have better emotion regulation compared to younger adolescents [75]. During early adolescence, a rise in emotional intensity may be caused by the maturation of affective centers in the brain (e.g., the amygdala), triggered by the release of gonadal hormones at the onset of puberty [80]. However, the cognitive control systems (e.g., the prefrontal cortex), which are associated with judgement and impulse control, usually matured later than the growing affective centers. This difference may explain the difficulty with emotional coping many younger adolescents have due to a lack of cognitive resources [80].

Furthermore, this study raises attention to the differences between senior high school and vocational high school students in Taiwan. Among males of the same age with low emotional eating, senior high school students were more likely than vocational high school students to frequently consume unhealthy foods. We found that students from different types of schools may be different in eating behaviors and other risk behaviors (data not shown). Previous studies observed different eating behaviors among high school students from different types of schools or educational contexts. Among Norwegian high school students, having an educational plan for university or college was positively associated with increased SSB consumption [81]. Other studies in Brazil [82] and Sri Lanka [83] showed differences in eating behaviors between private/international and federal/public high school students.

Similar to previous studies [38,39,40,41], our findings highlighted that frequent consumption of unhealthy foods, especially of SSBs, was common among Taiwanese adolescents. Commercial data from Taiwan revealed a very high concentration of convenience stores and beverage shops. According to the Taiwan Ministry of Economic Affairs, in 2019, the island had more than 11,100 convenience stores, which represented a ratio of one store for every 2125 people [84]. The density of beverages stores (including juice shops, beverage shops, coffee shops, and alcoholic beverage shops) was twice that of convenience stores [40]. In this context, public health interventions, such as policy making, should aim to promote nutrition label reading among young adolescents and provide healthier food choices at retail stores.

This study has several methodological limitations. First, since this was a cross-sectional study, the analyses conducted cannot prove the causal effect of emotional eating on the frequency of unhealthy food consumption. Second, this study used a novel approach to investigate emotional eating. Previously, emotional eating was measured by the Emotional Eating Scale in Children and Adolescents [34] or the Positive–Negative Emotional Eating Scale [85]. Nevertheless, the questions in our study about emotional eating frequency showed good internal consistency. Third, we did not investigate other confounders, such as negative body image, self-perception of weight, or sleep quality [59,72]. Additionally, the only socioeconomic determinant we considered for adjustment in our regression model was parental education. Parental education level can be used to reflect socioeconomic status. Family income is another indicator of socioeconomic status that should be adjusted in the regression model because a previous study demonstrated its association with UPFs consumption [20]. Unfortunately, however, our study lacks information on family income and family status, and this may be a limitation of our study. Finally, examining each unhealthy food group separately may lead to a misrepresentation of the effect of emotional eating on overall frequency of unhealthy food consumption.

Our study has several strengths. The nationwide sampling in this study provided a representative and consistent sample of the adolescent population in Taiwan. Moreover, our findings showed different health behaviors, including unhealthy food consumption among vocational high school students, senior high school, and junior high school students. When stratified by school types, adolescent populations had different lifestyles, social determinants, and harmful health behaviors such as smoking and binge drinking, all of which seem to be strong determinants of an adolescent’s unhealthy food consumption. The stratified results allow for the development of tailored health promotion interventions for each school type. Our adjusted model has controlled all the key confounders related to unhealthy food consumption.

## 5. Conclusions

This study demonstrated a positive association between emotional eating and frequent consumption of unhealthy food among adolescents in Taiwan. Eating while doing other activities and living a sedentary lifestyle were the two factors significantly associated with more frequent consumption of unhealthy foods across all five unhealthy food groups, while nutrition label reading was associated with less frequent consumption of unhealthy foods. Stratified analyses showed significantly different consumption patterns for unhealthy food groups among junior, senior, and vocational high school students across sex and emotional eating levels. Future longitudinal studies are recommended to better understand the causal pathway between emotional eating and unhealthy food consumption. A better understanding of the causal relationships would allow public health interventions to be more precisely targeted to prevent emotional eating and its negative health outcomes in young adolescents.

## Figures and Tables

**Table 1:** Characteristics of the participants and frequency of unhealthy food consumption.

Variable	Total (*n* = 18,461)	Male (*n* = 8953)	Female (*n* = 9508)	*p*
School type, *n* (%)				<0.001
Junior	6882 (37.3)	3572 (39.9)	3310 (34.8)	
Senior	4780 (25.9)	2059 (23.0)	2721 (28.6)	
Vocational	6799 (36.8)	3322 (37.1)	3477 (36.6)	
BMI, *n* (%)				<0.001
<18.5	5372 (29.7)	2546 (29.0)	2826 (30.3)	
≥24	3130 (17.3)	1910 (21.7)	1220 (13.1)	
18.5–24	9608 (53.0)	4330 (49.3)	5278 (56.6)	
Emotional eating, *n* (%)				<0.001
High	5841 (31.8)	2656 (29.8)	3185 (33.6)	
Low	12,546 (68.2)	6261 (70.2)	6285 (66.4)	
Fast food consumption, *n* (%)				<0.001
≥3 times/week	2948 (16.0)	1721 (19.2)	1227 (12.9)	
0–2 times/week	15,513 (84.0)	7232 (80.8)	8281 (87.1)	
High-fat snack consumption, *n* (%)				<0.001
≥3 times/week	4891 (26.5)	2580 (28.8)	2311 (24.3)	
0–2 times/week	13,570 (73.5)	6373 (71.2)	7197 (75.7)	
Processed meat product consumption, *n* (%)				<0.001
≥3 times/week	5504 (29.8)	3179 (35.5)	2325 (24.5)	
0–2 times/week	12,957 (70.2)	5774 (64.5)	7183 (75.5)	
Dessert food consumption, *n* (%)				<0.001
≥3 times/week	8599 (46.6)	3882 (43.4)	4717 (49.6)	
0–2 times/week	9862 (53.4)	5071 (56.6)	4791 (50.4)	
Sugar-sweetened beverage consumption, *n* (%)				<0.001
≥3 times/week	11,099 (60.1)	5789 (64.7)	5310 (55.8)	
0–2 times/week	7362 (39.9)	3164 (35.3)	4198 (44.2)	
Eating while doing something, *n* (%)				0.251
Yes	9660 (52.4)	4643 (52.0)	5017 (52.8)	
No	8767 (47.6)	4288 (48.0)	4479 (47.2)	
Nutrition label reading, *n* (%)				<0.001
Yes	8627 (46.9)	4019 (45.0)	4608 (48.6)	
No	9780 (53.1)	4909 (55.0)	4871 (51.4)	
Skipping breakfast, *n* (%)				<0.001
Yes	2077 (11.3)	930 (10.4)	1147 (12.1)	
No	16,376 (88.7)	8018 (89.6)	8358 (87.9)	
Sedentary activity, *n* (%)				0.003
Yes	10,000 (54.3)	4947 (55.4)	5053 (53.2)	
No	8425 (45.7)	3981 (44.6)	4444 (46.8)	
Physical activity, *n* (%)				<0.001
Yes	8564 (46.6)	5179 (58.2)	3385 (35.7)	
No	9806 (53.4)	3715 (41.8)	6091 (64.3)	
Binge drinking, *n* (%)				<0.001
Yes	1195 (6.5)	748 (8.4)	447 (4.7)	
No	17,254 (93.5)	8198 (91.6)	9056 (95.3)	
Smoking, *n* (%)				<0.001
Yes	1040 (5.6)	793 (8.9)	247 (2.6)	
No	17,411 (94.4)	8152 (91.1)	9259 (97.4)	
Peer support, *n* (%)				<0.001
Yes	15,773 (86.0)	7403 (83.4)	8370 (88.5)	
No	2562 (14.0)	1470 (16.6)	1092 (11.5)	
School support, *n* (%)				<0.001
Yes	16,832 (93.2)	7927 (90.7)	8905 (95.5)	
No	1231 (6.8)	811 (9.3)	420 (4.5)	
Father education, *n* (%)				0.850
University graduate	5951 (35.7)	2862 (35.6)	3089 (35.7)	
Senior high school graduate	7123 (42.7)	3447 (42.9)	3676 (42.5)	
Junior high school graduate and below	3613 (21.7)	1728 (21.5)	1885 (21.8)	
Mother education, *n* (%)				0.204
University graduate	5716 (34.1)	2771 (34.8)	2945 (33.6)	
Senior high school graduate	8186 (48.9)	3879 (48.7)	4307 (49.1)	
Junior high school graduate and below	2838 (17.0)	1321 (16.6)	1517 (17.3)	

**Table 2:** Association between personal factors and behavioral factors and frequent unhealthy food consumption.

Variables	Fast Foods		High-Fat Snacks		Processed Meat Products		Dessert Foods		SSBs	
	OR (95%CI)	*p*	OR (95%CI)	*p*	OR (95%CI)	*p*	OR (95%CI)	*p*	OR (95%CI)	*p*
Personal Factors										
Emotional Eating (High vs. Low)	2.40 (2.18–2.64)	<0.001	2.30 (2.12–2.49)	<0.001	1.92 (1.78–2.08)	<0.001	2.49 (2.31–2.69)	<0.001	1.83 (1.69–1.98)	<0.001
Sex (Male vs. Female)	1.63 (1.48–1.80)	<0.001	1.31 (1.21–1.42)	<0.001	1.71 (1.59–1.85)	<0.001	0.78 (0.73–0.84)	<0.001	1.43 (1.33–1.54)	<0.001
Behavioral Factors										
Eating while doing something (Yes vs. No)	2.05 (1.85–2.28)	<0.001	2.28 (2.09–2.47)	<0.001	1.72 (1.59–1.86)	<0.001	2.08 (1.94–2.28)	<0.001	2.17 (2.02–2.33)	<0.001
Label reading (Yes vs. No)	0.91 (0.83–0.99)	0.042	0.87 (0.80–0.94)	<0.001	0.93 (0.87–1.01)	0.068	0.97 (0.90–1.04)	0.356	0.82 (0.76–0.88)	<0.001
Sedentary activity (Yes vs. No)	1.59 (1.44–1.76)	<0.001	1.41 (1.30–1.53)	<0.001	1.33 (1.23–1.44)	<0.001	1.19 (1.11–1.28)	<0.001	1.62 (1.51–1.74)	<0.001
Binge drinking (Yes vs. No)	1.29 (1.09–1.53)	0.003	1.15 (0.98–1.34)	0.081	1.21 (1.04–1.40)	0.012	1.24 (1.07–1.44)	0.004	0.98 (0.84–1.15)	0.810
Smoking (Yes vs. No)	1.26 (1.05–1.51)	0.014	1.14 (0.97–1.36)	0.120	1.17 (0.99–1.38)	0.052	0.89 (0.76–1.05)	0.159	1.45 (1.22–1.74)	<0.001

OR: Odds ratio; CI: Confidence Interval; SSBs: sugar-sweetened beverages; Multivariate logistic regression models adjusted for BMI, school type, skipping breakfast, physical activity, peer support, school support, and parental education.

**Table 3:** Adjusted odds ratios of frequent unhealthy food consumption for different school types stratified by sex and emotional eating.

Sex	EmE	School Type	Fast Foods		High-Fat Snacks		Processed Meat Products		Dessert Foods		SSBs	
			OR (95%CI)	*p*	OR (95%CI)	*p*	OR (95%CI)	*p*	OR (95%CI)	*p*	OR (95%CI)	*p*
Male	High	Junior	1.04 (0.83–1.31)	0.750	0.77 (0.62–0.95)	0.015	1.24 (1.00–1.54)	0.045	0.92 (0.74–1.15)	0.475	0.98 (0.76–1.26)	0.884
		Senior	0.83 (0.64–1.07)	0.150	0.93 (0.74–1.18)	0.553	0.98 (0.78–1.24)	0.891	0.96 (0.75–1.22)	0.735	1.05 (0.78–1.38)	0.724
		V (ref)	1		1		1		1		1	
	Low	Junior	1.26 (1.03–1.55)	0.027	0.69 (0.58–0.81)	<0.001	1.15 (0.99–1.34)	0.064	0.78 (0.67–0.90)	0.001	1.16 (1.00–1.33)	0.046
		Senior	1.36 (1.09–1.70)	0.007	1.162 (0.98–1.39)	0.094	1.36 (1.16–1.60)	<0.001	1.18 (1.01–1.38)	0.035	1.47 (1.26–1.71)	<0.001
		V (ref)	1		1		1		1		1	
Female	High	Junior	1.04 (0.82–1.32)	0.735	0.83 (0.68–1.02)	0.072	1.08 (0.88–1.32)	0.460	0.91 (0.74–1.122	0.383	0.98 (0.79–1.21)	0.823
		Senior	0.89 (0.69–1.15)	0.377	0.86 (0.70–1.06)	0.165	1.05 (0.85–1.30)	0.651	1.06 (0.86–1.32)	0.569	0.81 (0.65–1.00)	0.054
		V (ref)	1		1		1		1		1	
	Low	Junior	1.11 (0.86–1.42)	0.430	0.76 (0.63–0.91)	0.003	0.89 (0.75–1.06)	0.192	0.63 (0.55–0.73)	<0.001	0.93 (0.81–1.07)	0.301
		Senior	1.00 (0.77–1.31)	0.984	0.93 (0.78–1.12)	0.453	1.04 (0.88–1.24)	0.629	1.05 (0.91–1.21)	0.487	1.09 (0.95–1.26)	0.222
		V (ref)	1		1		1		1		1	

OR: Odds ratio; CI: Confidence Interval; SSBs: sugar-sweetened beverages; V: vocational high school. Multivariate logistic regression model adjusted for BMI, eating while doing something, nutrition label reading, skipping breakfast, smoking, binge drinking, sedentary activity, physical activity, peer support, school support, and parental education.

## Data Availability

The data that support the findings of this study are available from the Taiwan Health Promotion Administration but are restricted for research use only. The data are not publicly available. Data are available from the authors upon reasonable request and with permission of the Taiwan Health Promotion Administration.
